# Surface Roughed and Pt-Rich Bimetallic Electrocatalysts for Hydrogen Evolution Reaction

**DOI:** 10.3389/fchem.2020.00422

**Published:** 2020-06-04

**Authors:** Fang Wang, Haifeng Yu, Ting Feng, Dan Zhao, Jinhua Piao, Jianfei Lei

**Affiliations:** ^1^School of Environmental Engineering and Chemistry, Luoyang Institute of Science and Technology, Luoyang, China; ^2^State Key Laboratory of Organic-Inorganic Composites, Beijing, China; ^3^School of Food Science and Engineering, South China University of Technology, Guangzhou, China; ^4^School of Physics and Engineering, Henan University of Science and Technology, Luoyang, China

**Keywords:** carbon nanotubes, dealloying, bimetallic, PtCo alloy nanoparticles, hydrogen evolution reaction

## Abstract

Platinum-based alloys with low cost transition metals have been considered as promising electrocatalysts in the field of sustainable energy conversion and storage. Herein, chloroplatinic acid, cobalt chloride, and carbon nanotubes are used as platinum, cobalt precursors, and carriers, respectively, to prepare rich Pt dealloying PtCo nanoparticles (SD-PtCo/CNT) via co-liquid phase reduction and chemical dealloying methods. The characterization and test results confirm that PtCo alloy nanoparticles are evenly dispersed on carbon nanotubes, further dealloying and resulting in the partial dissolving of cobalt, simultaneously generating a rich Pt layer and roughly active surface. Benefiting from the unique structure, the SD-PtCo/CNT catalyst displays obviously enhanced HER activity in both acidic and alkaline conditions. In 1.0 M KOH, SD-PtCo/CNT exhibits a low overpotential of 78 mV at 10 mA/cm^2^ and a small tafel slope (38.28 mV/dec). In 0.5 M H_2_SO_4_, SD-PtCo/CNT still shows the superior performance compared with un-dealloying PtCo/CNT, with an overpotential of 17 mV at 10 mA/cm^2^ and corresponding tafel slope of 21.35 mV/dec. The high HER activity of SD-PtCo/CNT can be attributed to the formation of a platinum rich layer and the uniformly dispersed PtCo nanoparticles supported on superior conductive carbon nanotubes, suggesting its great potential for hydrogen generation via water splitting.

## Introduction

Rapid economic growth has increased the demand for resources. As we all know, the development and utilization of fossil fuels have created a series of environmental problems which include energy exhaustion, climate change, and energy security. Therefore, there is a clear and urgent need to seek environmentally friendly and renewable energy sources which can be sustainably developed (Turner, 2004; Dresselhaus and Thomas, 2011). Among many clean energy sources, hydrogen attracts enormous attention and has been intensively studied and developed given its potential for environmentally friendly and sustainable uses.

However, the traditional methods of hydrogen production (such as methane steam reforming and coal gasification) can't really solve the problems of environmental pollution and CO_2_ emission (Khan et al., 2002; Armor, 2005). Driven by renewable energy, the electrolysis of water has become an attractive approach for hydrogen evolution (Wu et al., 2017; Zhao et al., 2018). Hydrogen evolution reaction (HER) is an important semi-reaction in electrochemical water splitting (Valenti et al., 2016), but it suffers from a serious issue of high overpotential in the process of electrolytic water for overcoming various resistance voltage drops and polarization electromotive forces in electrolytic cells. To reduce the energy consumption of water decomposition, it is necessary to develop an efficient catalyst which can reduce the overpotential of hydrogen evolution reactions.

Recently, non-Pt catalysts, including transition metal derivatives, such as phosphides, sulfides, and selenides or complexes (Yang et al., 2016; Henckel et al., 2017; Wang X. Y. et al., 2017; Yilmaz et al., 2017; Jing et al., 2018; Hua et al., 2019), especially metal organic frameworks (MOFs) derived micro/nanostructures (Lin et al., 2020; Wang Y. Z. et al., 2020) and single-atom catalysts (SACs), which are brought into our insight with enhanced HER performance (Lv et al., 2020; Pu et al., 2020), have attracted considerable research attention. Although the above novel materials have exhibited a good catalytic performance comparable to that of noble metals, the issue of long-term cycling stability and repeatability remains to be tackled. Platinum-based materials still play an important role in hydrogen evolution, both in acidic and alkaline solutions, especially in practical devices.

It is true that its high price and scarcity limit the application of platinum, which is inevitable (Rodriguez et al., 2014). Because of this, the researchers concentrate on exploring catalysts with low-content Pt to reduce the cost. One of the effective routes is to introduce secondary metals into precious metals, forming alloyed bimetallic complexes; this can not only decrease the content of Pt, but also adjust the electronic structure and active sites (Liu et al., 2011; Smiljanic et al., 2014). Further dealloying can enhance the utilization efficiency of precious metals in the alloyed complexes. A number of reported papers have testified that dealloying treatment based on noble and non-noble metal alloying material is an effective way to further improve the catalytic activity for Pt-based catalysts. It is worth noting that alloy metal nanoparticles can produce lattice strain, partly non-noble metals that are dissolved and generate enriched noble metal surface layers in the dealloying procedure (Strasser et al., 2010; Lu et al., 2015; He et al., 2016; Jana et al., 2016). It is important to note that dealloying of Pt-M (M: non-noble metal) alloy nanoparticle precursors can also engender nanostructured catalysts (Wang X. X. et al., 2020; Wu et al., 2020a,b).

Unfortunately, most current research on the application of dealloying catalysts are focused solely on ORR catalysis or the electrooxidation of small organic molecules (Qi et al., 2013; Wang F. et al., 2017). To date, the promising dealloying bimetallic catalysts are seldom developed and investigated in relation to hydrogen evolution in water splitting. Furthermore, the surface dealloying is generally implemented by an *in-situ* electrochemical dealloying method (Gasteiger et al., 2005; Mani et al., 2008; Gan et al., 2013; Han et al., 2015; Saquib and Halder, 2018), which is relatively complicated, has a high energy consumption or pollution emission, and is not suitable for real large-scale syntheses.

In this work, a simple and easy-to-implement *ex-situ* method for preparing robust surface dealloying PtCo bimetallic catalysts toward a hydrogen evolution reaction is reported. The outstanding carbon nanotubes were used as carriers, PtCo nanoparticles were uniformly deposited on carbon nanotubes by the co-liquid phase reduction method, ultimate surface dealloying catalyst, and SD-PtCo/CNT was obtained by HCl leaching. The dealloying scheme makes PtCo alloy surface roughening and generates a rich-Pt layer and more defect active sites. Benefiting from this surface effect, the as-prepared SD-PtCo/CNT is endowed with remarkable HER performance better than either the original alloy of PtCo/CNT or the commercial Pt/C, which means it holds promise of serving as the catalyst for hydrogen generation.

## Experimental Section

### Materials

Chloroplatinic (IV) acid hydrate (H_2_PtCl_6_·6H_2_O, ≥37.5% Pt), cobalt (II) chloride hexahydrate (CoCl_2_·6H_2_O, 98%), potassium hydroxide (pellets, 85%), and carboxyl group functionalized multiwall carbon nanotubes (MWCNT-COOH, 10–20 nm outer diameter, 0.5–2 μm length) were ordered from Sigma-Aldrich, Shanghai. Ethanol (extra pure, 99%) was bought from Acros Organics. Nafion solution (5 wt % solution in aliphatic alcohols and water) was obtained from Alfa Aesar by Thermo Fisher Scientific. Commercial Pt/C (20 wt%) was purchased from the Johnson-Matthey company. All reagents were used as purchased without any further treatment.

### Preparation of PtCo/CNT

H_2_PtCl_6_·6H_2_O (130.1 mg), CoCl_2_·6H_2_O (59.6 mg), and CNT (180.3 mg) were dissolved in turn into 500 mL of anhydrous ethanol with ultrasonic for 30 min, then put in an ice bath for about 5 min. NaBH_4_ (360 mg) which contained 100 mL anhydrous ethanol was poured into the previous solution and continuously stirred until well-distributed, this method was marked as the co-liquid phase reduction method. After that, the solution was centrifuged (4,000 rpm, 5 min) and washed with the mixture of ethanol and water (v/v = 4:1, 300 mL) several times. PtCo/CNT was dried under a vacuum at 50°C for 12 h and the platinum loading was controlled in 20%. The preparation scheme of Pt/CNT was the same as PtCo/CNT except for the addition of CoCl_2_·6H_2_O.

### Preparation of Dealloying PtCo/CNT (SD-PtCo/CNT)

The as-prepared PtCo/CNT (100 mg) was dispersed into 300 mL absolute ethanol with ultrasound for 10 min and subsequently put in an ice-water bath for another 5 min. In order to carry out the surface treatment procedure, the as-prepared 400 mL 0.5 M HCl-C_2_H_5_OH mixture solution was added into the above dispersion solution under stirring for 10 min. After being centrifuged (4,000 rpm, 5 min) and washed with the mixture of ethanol and water (v/v = 4:1, 300 mL) several times, the sample was allowed to dry under vacuum at 50 °C for 12 h. The SD-PtCo/CNT was finally obtained.

### Material Characterization

The crystallographic structure and phase purity of the nanoparticle samples was determined by X-ray powder diffraction (XRD, Bruker AXS D8 Advance) using a Cu Kα source (λ = 1.5406 Å) at 45 kV and 40 mA. The morphology characteristics and element mapping were examined by the high-resolution transmission electron microscopy (HRTEM, JEOL JEM 2100F) and high angle annular dark-field aberration-corrected scanning transmission electron microscopic (HAADF-STEM) in conjunction with an energy dispersive X-ray (EDX, Bruker QUANTAX). Both TEM and STEM were operated at a voltage of 200 kV.

### Electrochemical Measurements

All electrochemical experiments were conducted on a CHI760E and typical three-electrode system at room temperature. HER performance was consecutively measured in 0.5 M H_2_SO_4_ and 1.0 M KOH electrolyte. A graphite rod was used as counter electrode, and Ag/AgCl and Hg/HgO were used as reference electrodes in 0.5 M H_2_SO_4_ and 1.0 M KOH aqueous solution, respectively. The working electrode was constructed by loading 5 μL catalyst ink on a polished glassy carbon with a 3 mm diameter to obtain a catalyst loading about 0.35 mg cm^−2^. Generally, the catalyst ink was prepared by ultrasonically dispersing the 5 mg catalyst in a mix comprising of 495 μL ethanol and 495 μL DI-water with the adding of 10 μL Nafion solution (5 wt.%). Prior to each measurement, the acidic or basic electrolyte was bubbled with N_2_ saturation. Cyclic voltammetry (CV) measurements were conducted at 50 mV s^−1^ from −0.03 V to 1.22 V (vs. RHE) and 0.074V~1.274 V (vs. RHE) in acid and basic condition, respectively. Linear sweep voltammetry (LSV) measurements involved scanning at 5 mV s^−1^ from 0.12 V to −0.08 V (vs. RHE) in 0.5 M H_2_SO_4_ aqueous solution and 0.004 V ~ −0.186 V (vs. RHE) in 1.0 M KOH aqueous solution, respectively. Electrochemical impedance spectra (EIS) measurements were performed from 100 kHz to 0.1 Hz at the potential of 20 mV (vs. RHE) in 0.5 M H_2_SO_4_ aqueous solution and −76 mV (vs. RHE) in 1.0 M KOH aqueous solution. Chronamperometry tests were conducted at a potential of 10 mV (vs. RHE) in 0.5 M H_2_SO_4_ aqueous solution and −46 mV in 1 M KOH aqueous solution. The measured potentials vs. Ag/AgCl or Hg/HgO were calibrated relative to a reversible hydrogen electrode (RHE) scale. In acidic media, *E*_(RHE)_ = *E*_(Ag/AgCl)_+ 0.1989 V + 0.059 × pH and in alkaline media, *E*_(RHE)_ = *E*_(Hg/HgO)_ + 0.098 + 0.059 × pH. All tests were carried out with current-resistance (IR) compensation.

## Results and Discussion

### Material Synthesis and Characterization

**Figure 1:**
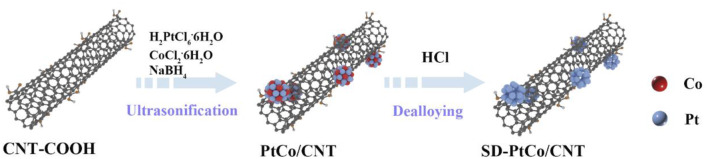
Schematic representation of the preparation process for surface dealloyed PtCo supported on CNT (SD-PtCo/CNT) catalyst.

The evenly distributed SD-PtCo/CNT was synthesized via co-liquid phase reduction and acid leaching, as shown in Figure 1. Carbon tubes were employed as a carrier to mix with chloroplatinic acid and cobalt chloride, then NaBH_4_ served as a reductant to fabricate PtCo/CNT. Further acid leaching meant Co was partially dissolved and exposed rich Pt and a roughed surface layer.

**Figure 2:**
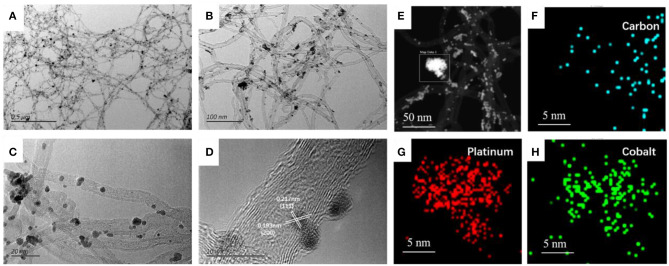
Typical TEM images **(A–C)** at different regions and different magnifications, HRTEM image **(D)** and HAADF-STEM image with the corresponding elementary mapping **(E–H)** of the as-prepared SD-PtCo/CNT.

The as-prepared surface dealloying PtCo alloy catalysts were first characterized using TEM, as shown in Figures 2A–D. The corresponding TEM images of the as-prepared PtCo/CNT and Pt/CNT were demonstrated in Figures S1, S2. It is interesting to note that the dealloying PtCo nanoparticles had better dispersion when compared with PtCo/CNT. The average particle size measured from randomly counting 100 particles was 2.5 and 2.3 nm for PtCo/CNT and SD-PtCo/CNT, respectively (Figure S3). It is basically consistent with the X-ray diffraction (XRD) results calculated using the (220) diffraction peak. Meanwhile, the higher magnification images revealed PtCo nano-particles appeared as lattice fringes, it was clearly identified from Figure 2D that the d-spacings for the nanoparticle were 0.138 and 0.187 nm, which corresponding to the (220) and (200) planes of face-centered cubic phase of PtCo alloy (JCPDS No. 43-1358) (Hu and Yu, 2013; Ren et al., 2018), indicating successful synthesis of the PtCo alloy. Furthermore, HAADF-STEM image and the corresponding EDX mapping of the SD-PtCo/CNT were depicted in Figures 2E–H, the carbon species were evenly distributed throughout the composites, which may mainly derive from carbon-based carrier CNTs. The Pt and Co elements also were homogeneously overlaid and had no obvious reunion, and these are in good agreement with the results observed from HRTEM images. The distribution positions of Pt were not exactly similar to Co, which indicated that the addition of HCl partly dissolved Co of the PtCo alloy and exposed more active Pt sites to form a rich Pt layer. This result is wholly consistent with other reported dealloying Pt-M (M: transition metal) (Shreya et al., 2017; Saquib and Halder, 2018).

**Figure 3:**
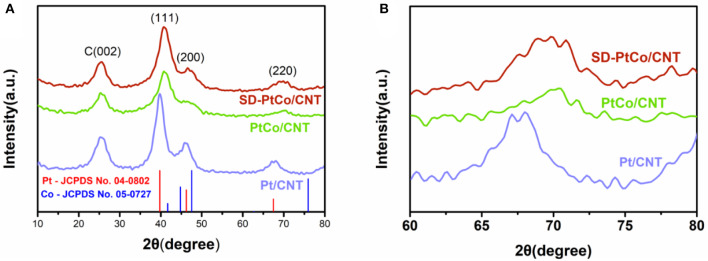
XRD patterns **(A)** and the enlarged view **(B)** of the as-prepared SD-PtCo/CNT, PtCo/CNT, and Pt/CNT.

The successful synthesis of PtCo alloy and the formation of a rich Pt layer after dealloying were also verified by XRD. The characteristic peak of carbon centered at 25.16° was observed in Figures 3A,B, and could be ascribed to carbon nanotubes. The typical diffraction peaks of SD-PtCo/CNT which emerged at 40.82, 46.76, and 70.16° were associated with the (111), (200), and (220) planes of the face-centered cubic PtCo alloys, respectively. This was consistent with TEM and STEM-EDX results. The diffraction peaks of Pt/CNT and PtCo/CNT emerged at 25.16, 39.56, 46.58, 68.18 and 25.16, 40.82, and 70.34°, respectively. It was noted that the diffraction peaks gradually shifted to a low angle from PtCo/CNT to Pt/CNT which demonstrates the formation of the PtCo alloy. There is no peak shift between SD-PtCo/CNT and PtCo/CNT. It implies that the dealloying treatment can keep the PtCo alloy skeleton structure, resulting in the synergistic effect of bimetal which is favorable to electrocatalysis. On the other hand, the Co atoms on the surface of the PtCo alloy were moved and the Pt atoms in the sub-layer were exposed by dealloying endowing the as-prepared catalyst a Pt-rich and rough surface (Saquib and Halder, 2018).

**Figure 4:**
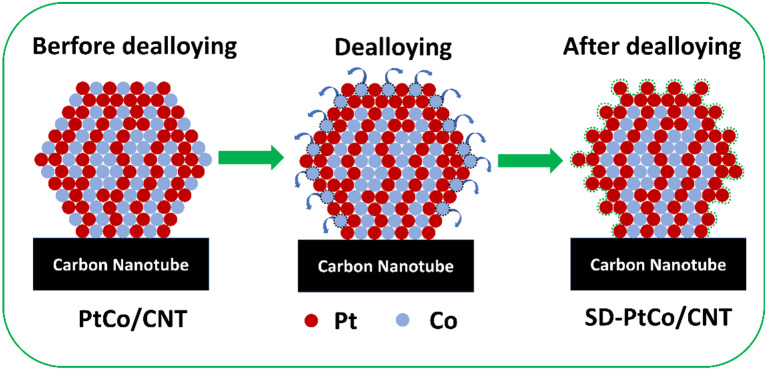
Schematic diagram of atom distribution before and after dealloying of PtCo alloy nanoparticles supported on carbon nanotube.

Figure 4 vividly revealed the distribution of metal atoms before and after the dealloying of the PtCo catalyst. It demonstrated that Co on the surface was more abundant than of Pt and exposed more Pt active sites. Meanwhile, the surface of the PtCo/CNT catalyst becomes rougher after dealloying due to the absence of the Co atoms. It is foreseeable that more active and rougher surfaces contain more Pt sites incorporated with a certain number of defects generated through dealloying. The STEM line scanning of a few nano-particles in the SD-PtCo/CNT catalyst further supported this speculation. As was shown in Figures S4, S5, it can be seen that there are fewer Co atoms on the surface of the dealloying PtCo catalyst while more Pt atoms are exposed through the obversion of Pt-L and Co-K signals in the line scanning. Furthermore, the corresponding EDX results provided the atomic ratio of the surface PtCo in the catalyst of SD-PtCo/CNT and PtCo/CNT (Figure S6). After dealloying, the PtCo atomic ratio increased from 64.6:35.4 to 70.6:29.4, which is in agreement with the ICP-AES results (Listed in Table S1), confirming the rough and Pt-rich surface by the moving of surface Co atoms.

### Electrocatalytic HER Performance of Materials

**Figure 5:**
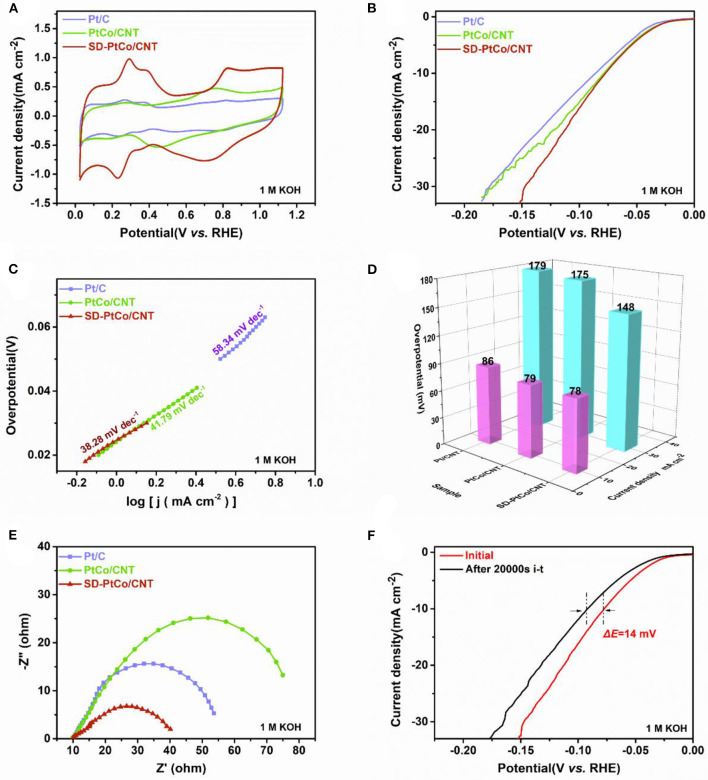
**(A)** Cyclic voltammetry curves of SD-PtCo/CNT, PtCo/CNT, and Pt/C. **(B)** HER polarization curves at 5 mV/s for SD-PtCo/CNT, PtCo/CNT, and Pt/C. **(C)** Corresponding tafel plots for the samples. **(D)** Corresponding overpotential of the samples at current density 10 mA cm^−2^ and 30 mA cm^−2^. **(E)** EIS Nyquist plots obtained at overpotential −76 mV and frequency range of 0.1–10^5^ Hz. **(F)** Polarization curves of the SD-PtCo/CNT obtained initial and after 20000s i-t (All the above tested in 1.0 M KOH).

The stability and activity of as-prepared materials were investigated to evaluate HER performance. It has been confirmed that the good activity is derived from the electrochemically active area (ECSA) of the catalyst normally determined by the hydrogen adsorption-desorption peaks of the cyclic voltammograms (CVs). Figure 5A depicted CVs of the as-prepared SD-PtCo/CNT, PtCo/CNT, and commercial Pt/C. It can be seen that the SD-PtCo/CNT exhibited the largest ECSA which was mostly ascribed to the highly active and roughed surface. The LSV test in a typically three-electrode system revealed the intrinsic electrochemical behavior of materials. As expected, Figures 5B,D showed the SD-PtCo/CNT had a relatively small initial overpotential of 78 mV at the current density of 10 mA/cm^2^, while they were 79 mV and 86 mV for Pt/C and PtCo/CNT, respectively. Simultaneously, the overpotential of 148 mV was observed for SD-PtCo/CNT at 30 mA/cm^2^ which is still lower than other control materials. The results implied the subtle role of sub-layer Co and the dealloying process in enhancing the HER performance. Tafel plots were obtained from LSV curves fitting, and were further given the electrocatalytic mechanism for exploring remarkable activity (Figure 5C). The tafel slopes of 38.28 and 41.97 mV/dec for SD-PtCo/CNT and PtCo/CNT were observed, slightly smaller than that of commercial Pt/CNT which is 58.34 mV/dec. The results indicated that the SD-PtCo/CNT showed rapid catalytic kinetics during the reaction of hydrogen evolution, which might be mainly dependent on the (Volmer-Heyrovsky Conway and Tilak, 2002; Liu et al., 2018). Previous reports indicated that when using the hydrochloride to remove Co or Cu from a Pt-M alloy, it may lead to decreased Pt-Pt interatomic distances and cause Pt d-bands to downshift, resulting in the strength of metal-adsorbate bond weakening. Thus, hydrogen was easier to be desorbed from Pt surface, which was beneficial to the reaction kinetic of HER (Saquib and Halder, 2018). Meanwhile, the enlarged ECSA result from the roughed surface and more exposed Pt active sites incorporated with a certain number of defects in the dealloying PtCo catalyst also contributed to the performance improvement.

To further evaluate the nature of electrocatalytic interfacial reaction kinetics, the EIS techniques of as-prepared catalysts were used in 1 M KOH. As shown in Figure 5E, the SD-PtCo/CNT exhibited the smallest diameter of impedance arc, when compared with PtCo/CNT and Pt/C. The results revealed that the SD-PtCo/CNT possessed the smallest charge transfer resistance and rapid proton reduction kinetics. This could be because the addition of Co adjusted the electron structure and active sites. Besides, the procedure of dealloying generated a rough and enriched-Pt surface and exposed more Pt, accordingly improved the utilization rate of Pt and enhancing the electrocatalytic performance. The long-term stability of catalysts was also assessed by i-t curves. The electrode was operated at −46 mV in 1 M KOH, a slight drop in current density after 20000 s i-t test can be observed in Figure 5F. Before and after i-t test, the difference of overpotential was about 14 mV at 10 mA cm^−2^; this change was insignificant. This demonstrated the good stability of SD-PtCo/CNT during the HER process, which is ascribed to the superiority of the PtCo alloy.

**Figure 6:**
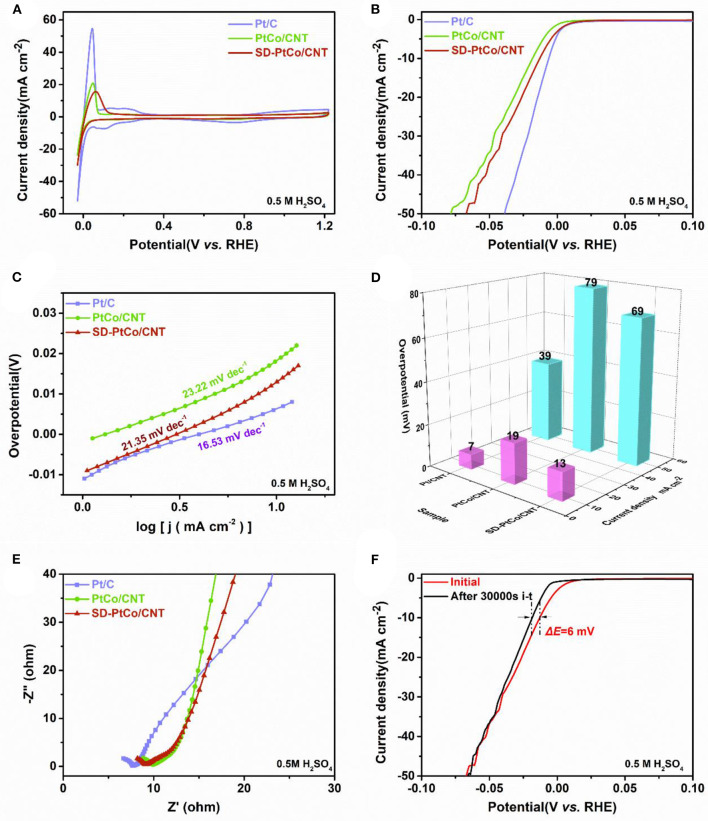
**(A)** Cyclic voltammetry curves of SD-PtCo/CNT, PtCo/CNT, and Pt/C. **(B)** HER polarization curves at 5 mV/s for SD-PtCo/CNT, PtCo/CNT, and Pt/C. **(C)** Corresponding tafel plots for the samples. **(D)** Corresponding overpotential of the samples at current densities of 10 and 50 mA cm^−2^. **(E)** EIS Nyquist plots obtained at overpotential 20 mV and frequency range of 0.1–10^5^ Hz. **(F)** Polarization curves of the SD-PtCo/CNT obtained initially and after 30000s i-t (All the above tested in 0.5 M H_2_SO_4_).

Electrocatalytic activity of SD-PtCo/CNT toward HER in acid electrolyte was also carried out. Figures 6A,B depicted CVs and LSVs of the as-prepared SD-PtCo/CNT, PtCo/CNT, and commercial Pt/C determined in 0.5 M H_2_SO_4_. The typical overpotential at certain current densities read from LSVs were simultaneously graphed in Figure 6D. As it was shown, the overpotential of SD-PtCo/CNT were 13 mV and 69 mV at a current density of 10 and 50 mA cm^−2^, respectively. Meanwhile, the corresponding tafel slope of SD-PtCo/CNT was 21.35 mV/dec (Figure 6C). As compared with PtCo/CNT, the surfaced dealloyed PtCo catalyst had a lower tafel slope revealing the rapid rate of hydrogen production and reaction kinetics. But the dealloying SD-PtCo/CNT catalyst, as well as PtCo/CNT, demonstrated a higher tafel slope as compared with the commercial Pt/C in acid condition. This may be caused by the electrochemical corrosion of the acid electrolyte. As for PtCo/CNT, it was existing surface dealloying by removing the Co sites like SD-PtCo/CNT, but greater electrochemical corrosion should be occurred in such strong acid of H_2_SO_4_ electrolyte. As for SD-PtCo/CNT, the effect of the electrochemical corrosion resulted in a greater electrochemical polarization due to the roughed surface. Figure 6E displayed the Nyquist plots of the synthetic catalysts. Obviously, commercial Pt/C exhibited the smallest diameter of impedance arc, following an order of Pt/C < SD-PtCo/CNT < PtCo/CNT. In comparison with un-dealloying PtCo/CNT, the SD-PtCo/CNT suggested faster proton reduction kinetics and charge transfer resistance. As far as we know, the stability of HER catalysts was a most important parameter of catalytic performance. Therefore, the long-term stability of different catalysts was further investigated. Different from the alkaline electrolyte, the general catalyst exhibits a relatively good long-term stability in acid condition because more H^+^ are available in acid electrolytes, accelerating the reaction kinetics. As shown in Figure 6F, after 30000 s i-t test, the overpotential of SD-PtCo/CNT only changed 6 mV at 10 mA cm^−2^, which also demonstrates the effect of electrochemical corrosion to a certain extent.

Combining the above results, the SD-PtCo/CNT displayed excellent HER activity and stability as compared with PtCo/CNT, which is due to the unique rough Pt-rich surface shell and PtCo alloy core caused by the dealloying treatment. Firstly, the chemically dealloying process not only generated a roughed and Pt-rich surface with more active Pt sites exposed, but also formed an amount of defect sites, which maximized the utilization of Pt, reduced the amount of Pt, and boosted the HER performance. Secondly, the sub-layer Co has a certain catalytic for hydrogen evolution, resulting in enhancing the catalytic performance of the material. The introduction of cobalt also changed the electron structure and active sites. Finally, the performance improvement comes from the carrier effect. The CNTs not only improved the conductivity of the material, but also enhanced the dispersion of nanoparticles.

## Conclusions

In summary, we have demonstrated a simple co-liquid phase reduction synthetic strategy for the preparation of PtCo/CNT and a successful design of surface dealloying material, SD-PtCo/CNT through HCl leaching. The results showed that carbon nanotubes not only improved the conductivity of materials, but also effectively hindered the agglomeration of alloy nanoparticles. Simultaneously, the dealloying procedure of PtCo/CNT fabricated rough and rich-Pt layers and meant more Pt was exposed to the surface; the outstanding performance test results of HER in acidic and alkaline are enough to prove that. The lower overpotential of SD-PtCo/CNT were 78 and 13 mV at 10 mA cm^−2^ in 1 M KOH and 0.5 M H_2_SO_4_, respectively. This simple and easy to realize dealloying method provides new direction for the fabrication of high performance HER catalyst and other alloy materials.

## Data Availability Statement

The raw data supporting the conclusions of this article will be made available by the authors, without undue reservation, to any qualified researcher.

## Author Contributions

FW have made substantial contributions to the design of this work and have drafted the work. HY have revised the work critically for important intellectual content and have approved the final version to be published. TF have made substantial contributions to the acquisition, analysis, and interpretation of data for the work. DZ and JP have made some contributions to the analysis and interpretation of data for the work. JL have provided some important ideas to the design of this work and have approved the final version to be published.

## Conflict of Interest

The authors declare that the research was conducted in the absence of any commercial or financial relationships that could be construed as a potential conflict of interest.
