# Supplementary material for: Surface Roughed and Pt-Rich Bimetallic Electrocatalysts for Hydrogen Evolution Reaction
Source: Front Chem. 2020 Jun 4;8:422. doi: 10.3389/fchem.2020.00422 (PMC7287206; doi:10.3389/fchem.2020.00422)
Supplement: Supplementary file 1 [file Table_1.docx]

**Supporting Information**

**Surface Roughed and Pt-rich Bimetallic Electrocatalysts for Hydrogen Evolution Reaction**

***Fang Wang^1,3^, Haifeng Yu^1*^, Ting Feng^1^, Dan Zhao^1^, Jinhua Piao^4^, Jianfei Lei^2**^***

*^1^ School of Environmental Engineering and Chemistry, Luoyang Institute of Science and Technology, Luoyang 471023, China, ^2^ School of Physics and Engineering, Henan University of Science and Technology, Luoyang 471023, China, ^3^ State Key Laboratory of Organic-Inorganic Composites, Beijing 100029, China, ^4^ School of Food Science and Engineering, South China University of Technology, Guangzhou 510641, China*

****Correspondence:***

*Haifeng Yu*

*yhf1116@163.com*

*Jianfei Lei*

[*leijianfei9966@163.com*](mailto:leijianfei9966@163.com)


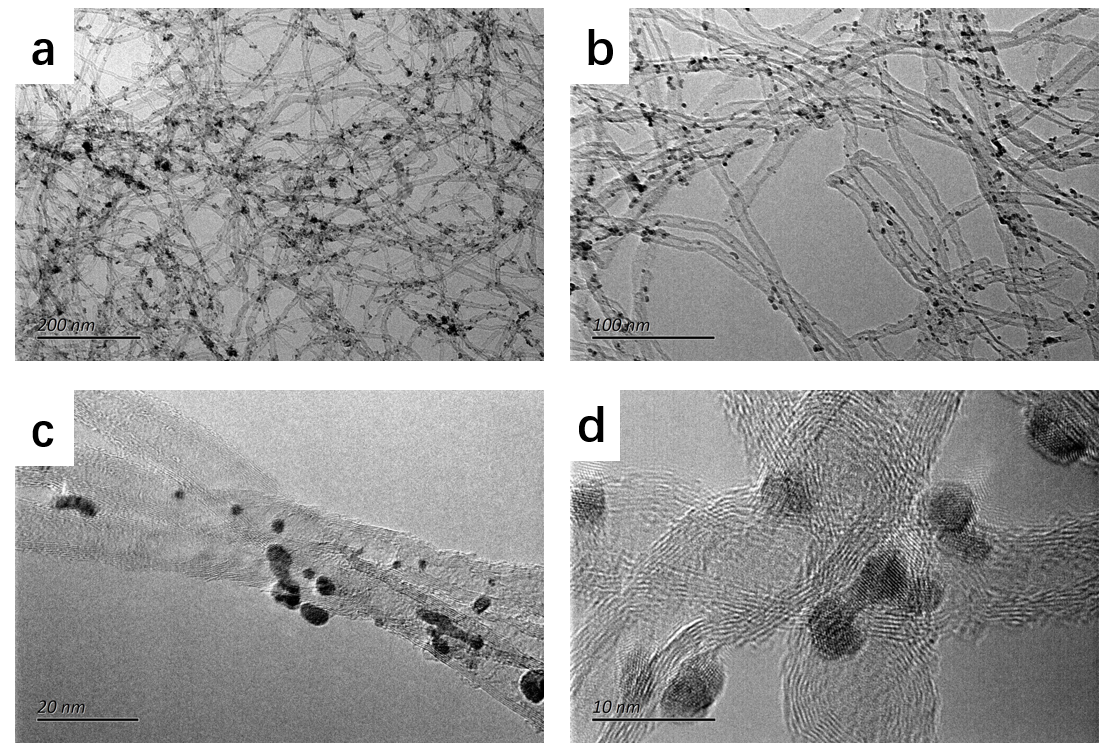


**Figure S1 Typical TEM images(a-c) at different regions and different magnifications， HRTEM image(d)** **of the as-prepared PtCo/CNT.**

**
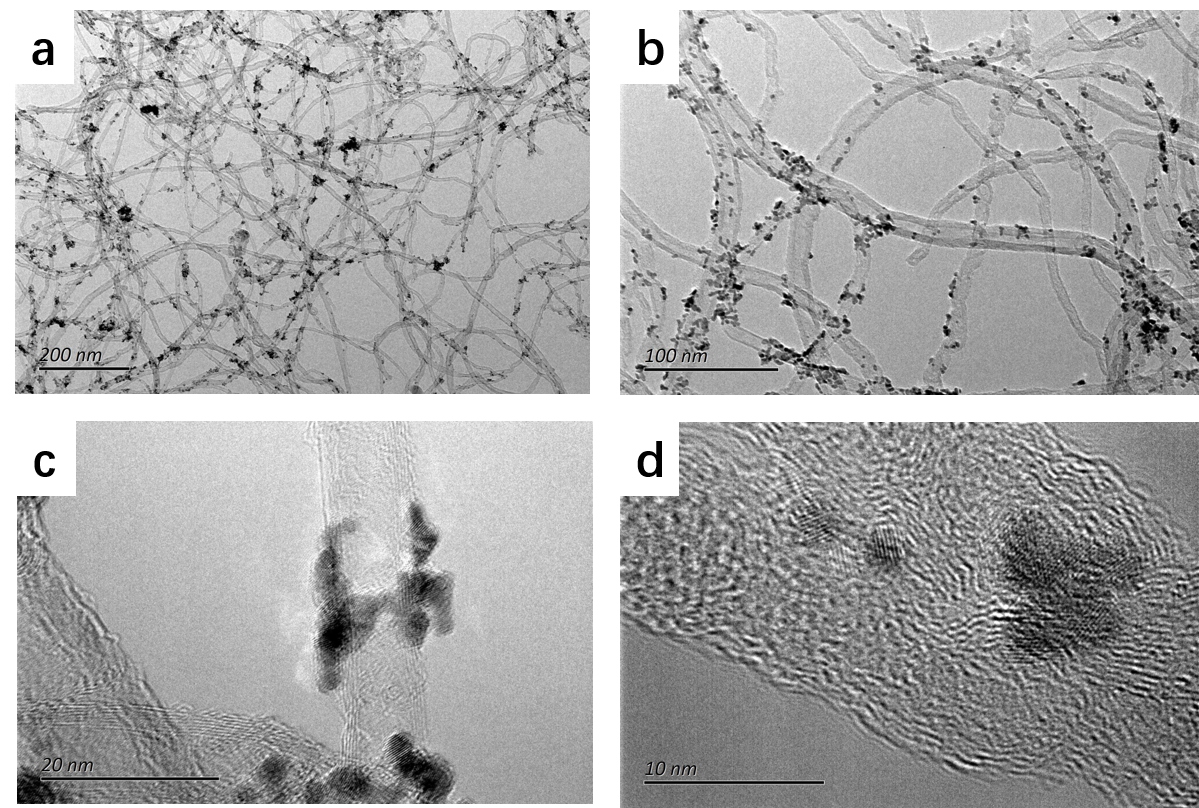
**

**Figure S2 Typical TEM images(a-c) at different regions and different magnifications， HRTEM image(d) of the as-prepared Pt/CNT.**

**
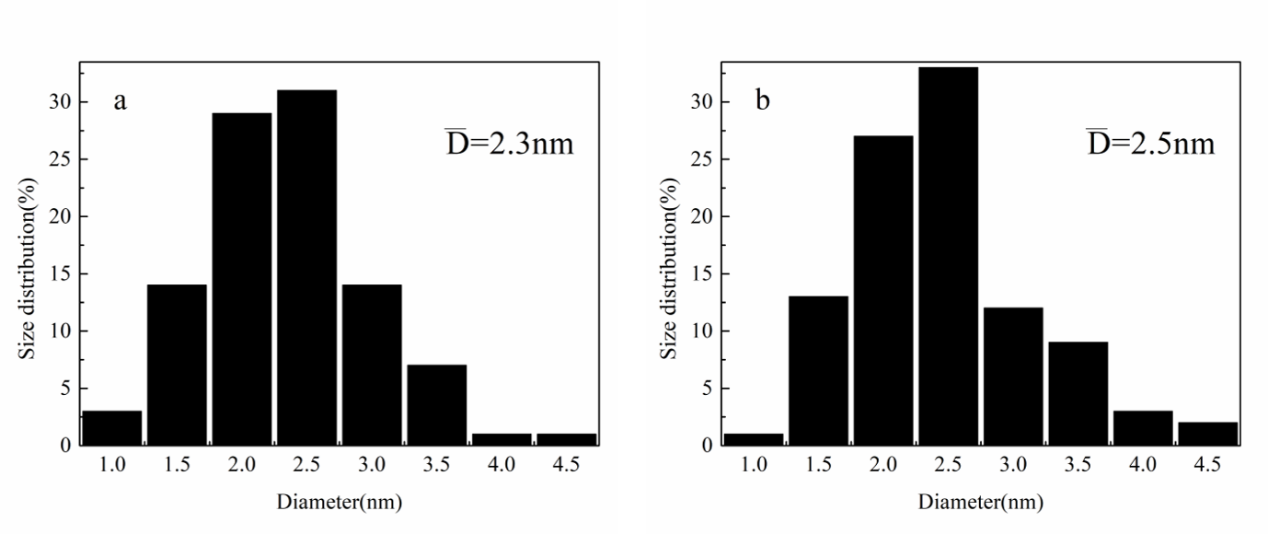
Figure S3 Particle size distributions of the as-prepared SD-PtCo/CNT (a) and PtCo/CNT (b)**

**
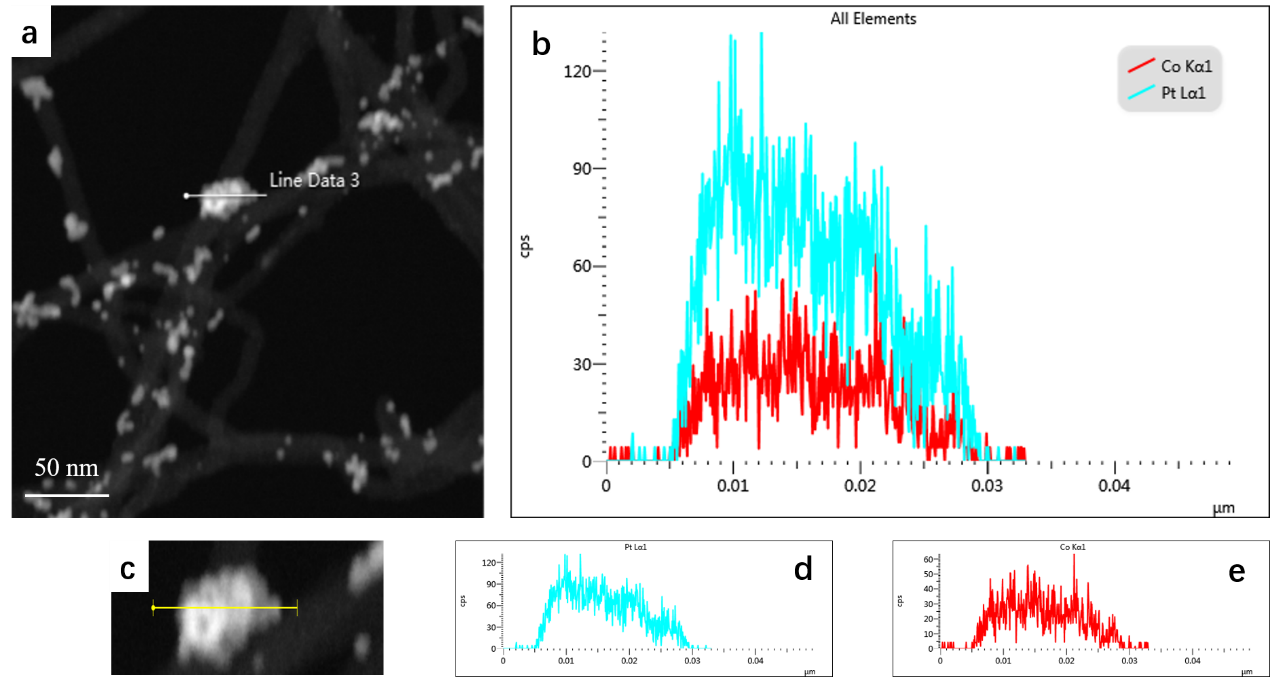
**

**Figure S4 HAADF-STEM Line scanning of the PtCo nano-particles in the as-prepared SD-PtCo/CNT catalyst.**

**
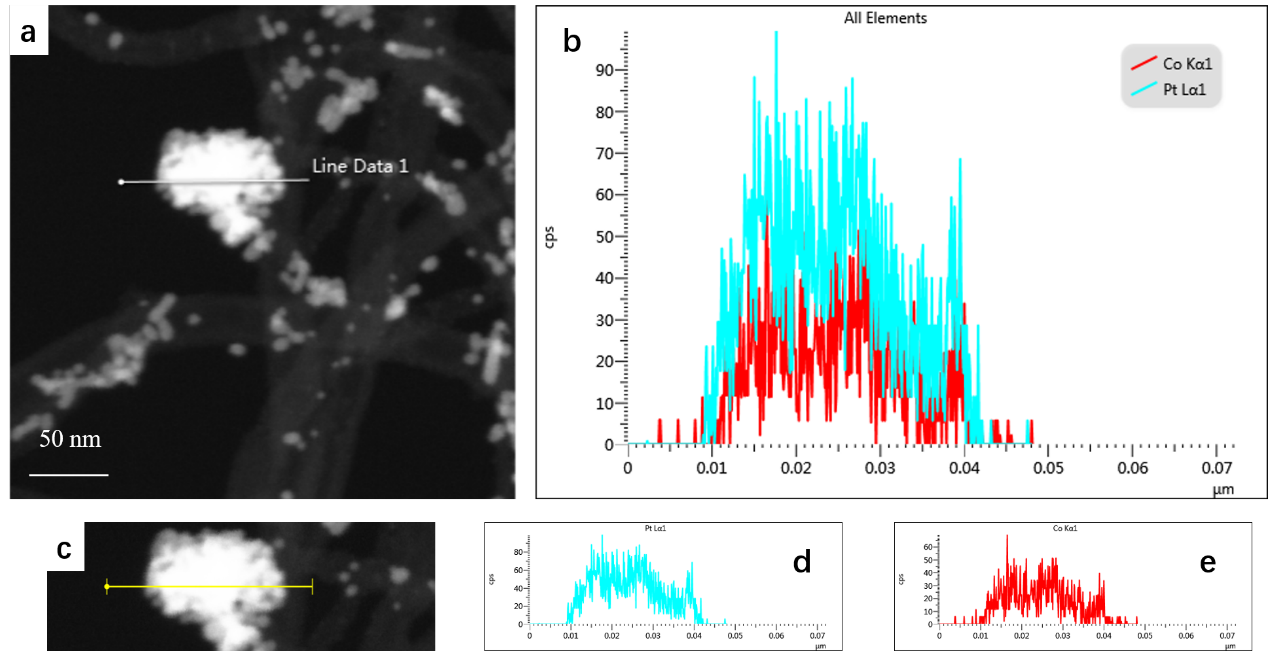
**

**Figure S5 HAADF-STEM Line scanning of the PtCo nano-particles in the as-prepared PtCo/CNT catalyst.**

**
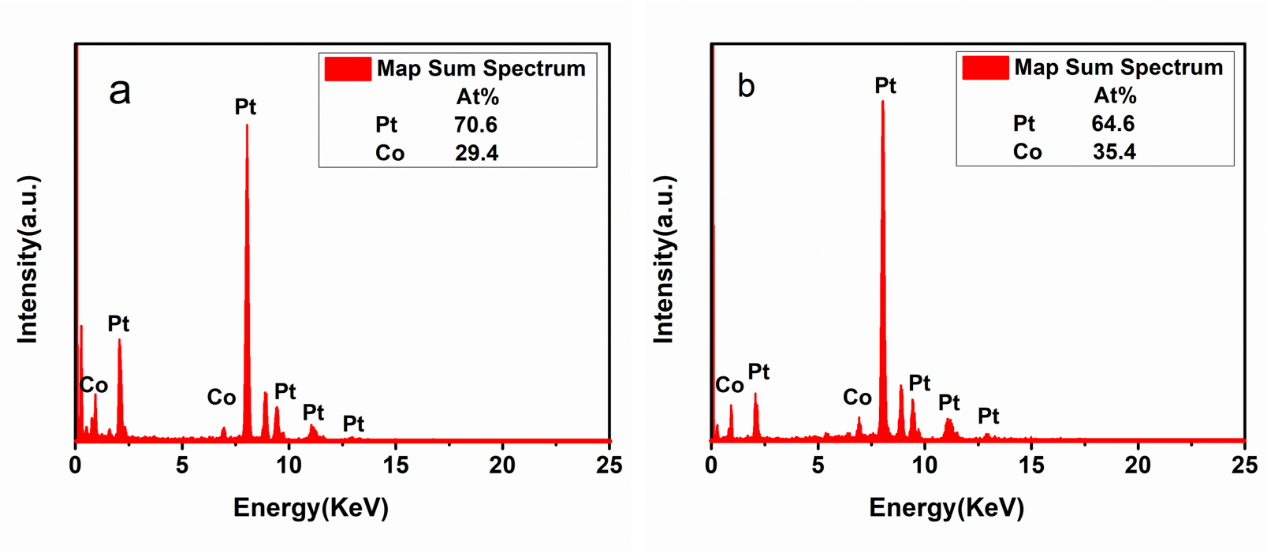
**

**Figure S6 Energy dispersive X-ray Spectroscopy of the as-prepared SD-PtCo/CNT (a) and PtCo/CNT (b).**

**Table S1 The loading and elemental compositions of as-prepared SD-PtCo/CNT and PtCo/CNT determined by ICP-AES (catalysts were dissolved in aqua regia solution (‘royal water’) over night before the test).**

| Catalyst | Pt loading (wt%) | Pt:Co (Atomic Ratio) |
| --- | --- | --- |
| PtCo/CNT | 19.2 | 1.88 |
| SD-PtCo/CNT | 19.8 | 2.48 |
